# Removal of calciprotein particles from the blood using an adsorption column improves prognosis of hemodialysis miniature pigs

**DOI:** 10.1038/s41598-023-42273-0

**Published:** 2023-09-12

**Authors:** Marina Miura, Yutaka Miura, Yoshitaka Iwazu, Hideyuki Mukai, Takahiro Sugiura, Yuji Suzuki, Masami Kato, Mayumi Kano, Daisuke Nagata, Kazuhiro Shiizaki, Hiroshi Kurosu, Makoto Kuro-o

**Affiliations:** 1 Division of Anti-Aging Medicine, Center for Molecular Medicine, Jichi Medical University, 3311-1 Yakushiji, Shimotsuke, Tochigi 329-0498 Japan; 2 Department of Clinical Laboratory Medicine, Jichi Medical University, Tochigi, Japan; 3 Division of Nephrology, Department of Internal Medicine, Jichi Medical University, Tochigi, Japan; 4 Nihon Bioresearch Inc., Gifu, Japan

**Keywords:** Haemodialysis, Calcium and phosphate metabolic disorders

## Abstract

Hyperphosphatemia is a major risk for poor prognosis in patients with end-stage renal disease. However, the molecular mechanism behind this link remains elusive. We and others have demonstrated that serum phosphorus levels correlate positively with circulating levels of calciprotein particles (CPPs). CPPs are colloidal mineral-protein complexes containing insoluble calcium-phosphate precipitates and have been reported to induce calcification in cultured vascular smooth muscle cells and inflammatory responses in cultured macrophages. Hence, we hypothesize that CPPs may be responsible for disorders associated with hyperphosphatemia. Using hyperphosphatemic miniature pigs receiving hemodialysis, here we show that removal of CPPs from the blood with a newly developed CPP adsorption column improves survival and alleviates complications including coronary artery calcification, vascular endothelial dysfunction, metastatic pulmonary calcification, left ventricular hypertrophy, and chronic inflammation. The present study identifies CPPs as an effective therapeutic target and justifies clinical trials to determine whether the CPP adsorption column may be useful as a medical device for improving clinical outcomes of hemodialysis patients.

## Introduction

Calciprotein particles (CPPs) are defined as mineral-protein complexes containing insoluble calcium-phosphate and serum protein fetuin-A^1–12^. CPPs are formed spontaneously in the solution containing phosphate, calcium, and serum^5,13,14^. When the phosphate and calcium concentration exceed the solubility limit, amorphous (non-crystalline) calcium-phosphate precipitates appear in the solution. These precipitates are adsorbed by fetuin-A to form single fetuin-A molecules loaded with amorphous calcium-phosphate precipitates, which are called calciprotein monomers (CPMs). CPMs undergo self-aggregation over time and grow into nanoparticles with a diameter of 30 ~ 100 nm, which are designated as primary CPPs. Primary CPPs further agglomerate and grow into larger particles with a diameter of a few hundred nm when the calcium-phosphate precipitates undergo amorphous-to-crystalline phase transition. CPPs containing crystalline calcium-phosphate are designated as secondary CPPs (Supplementary Fig. S1a). Secondary CPPs induce calcification when applied to cultured vascular smooth muscle cells^15^. Both primary and secondary CPPs can induce innate immune responses when applied to cultured macrophages^16–18^.

CPPs appear in the blood. Recent clinical studies identified blood phosphate concentration as a major and independent determinant of the circulating CPP level^2,3^. Serum phosphate and CPP levels are increased in patients with progression of chronic kidney disease (CKD)^2,3,19^. Furthermore, serum CPP levels were reported to correlate positively with clinical parameters for vascular calcification (coronary artery calcification score), aortic stiffness (aortic pulse wave velocity), and inflammation (C-reactive protein and cytokines) in CKD patients^2,18–20^. Because hyperphosphatemia is a major risk for vascular calcification and poor prognosis^21^, we hypothesize that CPPs may be a causative agent of poor clinical outcomes associated with hyperphosphatemia in end-stage renal disease patients.

To test this hypothesis, we developed a CPP adsorption column. CPPs are not removed by regular hemodialysis because CPPs are too large to pass through the dialysis membrane. The CPP adsorption column was filled with cellulose beads conjugated with alendronate, a bisphosphonate that bound to calcium-phosphate crystals^3^. The alendronate column was inserted into the hemodialysis circuit to capture and remove CPPs from the circulation during hemodialysis sessions. We performed a pre-clinical study to test effectiveness of the alendronate column using miniature pigs.

## Results

**Figure 1:**
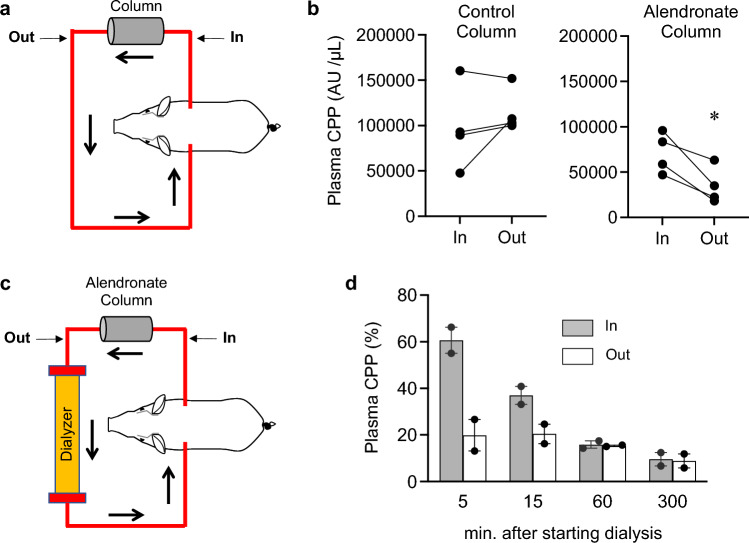
Removal of circulating CPPs using the alendronate column in nephrectomized miniature pigs. (**a**) A schematic representation of extracorporeal circulation using the alendronate column or the control column. Blood sampling was performed at the inlet (In) and the outlet (Out) of the column 30 min after starting the extracorporeal circulation. Plasma CPP levels were determined by the gel-filtration method^3^. (**b**) Difference in the plasma CPP levels between the inlet and the outlet. The alendronate column, but not the control column, adsorbed CPPs. *N* = 4 for each group, *P = 0.028 by paired t-test. (**c**) A schematic representation of the hemodialysis circuit using the alendronate column. (**d**) Difference in the plasma CPP levels between the inlet (In) and the outlet (Out) of the column at the indicated time points after starting hemodialysis. The data from two pigs were expressed as percent of the CPP levels before starting hemodialysis (median with range).

First, we evaluated the ability of the alendronate column to remove CPPs from the blood. On the 4th day after bilateral nephrectomy, miniature pigs were subjected to extracorporeal circulation using either the alendronate column or a control column filled with cellulose beads without alendronate conjugation (Fig. 1a). CPP levels were quantified in the blood samples collected from the inlet and the outlet of the columns by the gel-filtration method, which principally measured the amount of crystalline calcium-phosphate in secondary CPPs^3^. The CPP levels at the outlet were significantly lower than those at the inlet in the alendronate column, but not in the control column (Fig. 1b), indicating that the alendronate column removed secondary CPPs from the blood.

We then evaluated the CPP adsorption capacity of the alendronate column when inserted into the hemodialysis circuit (Fig. 1c). Nephrectomized miniature pigs were maintained by hemodialysis every other day. Blood samples were collected from the inlet and the outlet of the alendronate column 5, 15, 60, and 300 min after starting hemodialysis to measure CPP levels by the gel-filtration method. Again, reduction of CPP levels was observed at the outlet within the first 15 min (Fig. 1d), indicating that the alendronate column had sufficient capacity for removing CPPs from the circulation during hemodialysis sessions.

### Survival

**Figure 2:**
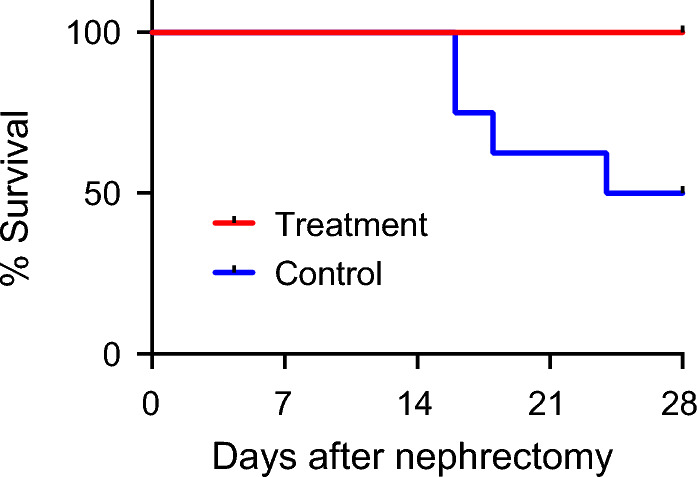
Effects of the alendronate column on mortality. Kaplan–Meier survival curves of the alendronate group (red, *N* = 8) and the control group (blue, *N* = 8). *P* = 0.0247 by log-rank test.

After bilateral nephrectomy, miniature pigs were assigned randomly to maintenance hemodialysis every other day using either the alendronate column (the treatment group; *N* = 8) or the control column (the control group; *N* = 8) in every session. Despite the similar hemodialysis efficiency as determined by Kt/V (Table 1), all the 8 pigs in the treatment group, whereas only 4 out of the 8 pigs in the control group, survived for 28 days of the observation period (*P* = 0.025 by log-rank test, Fig. 2). The pigs were dissected at the end of the observation period or at ethical endpoints. Heart failure or sudden cardiac death seemed the most likely cause of death in the control group, because pulmonary edema was evident but cerebral infarction/hemorrhage or myocardial infarction were not observed at autopsy.

**Table 1:** Results of clinical tests performed on Day 0 and Day 14.

Parameters	Control (*N* = 8)		Treatment (*N* = 8)		Control vs. treatment on day 14
	Day 0	Day 14	Day 0	Day 14	*P*
Systolic blood pressure (mmHg)	111 ± 22	109 ± 32	137 ± 21	130 ± 24	0.170
Diastolic blood pressure (mmHg)	83 ± 18	59* ± 23	95 ± 21	81 ± 21	0.072
Pulse pressure(mmHg)	28 ± 13	50** ± 12	42 ± 14	49 ± 13	0.896
Heart rate(beats/min)	122 ± 27	138 ± 20	105 ± 13	109 ± 35	0.059
pH	7.465 ± 0.060	7.390* ± 0.051	7.502 ± 0.049	7.380** ± 0.029	0.653
pCO_2_ (mmHg)	45.6 ± 7.4	32.9** ± 2.9	41.0 ± 8.4	33.6* ± 3.2	0.682
pO_2_ (mmHg)	87.4 ± 13.2	78.3 ± 8.8	85.3 ± 11.3	90.8 ± 14.4	0.055
Base excess (mmol/L)	8.6 ± 2.2	− 4.8** ± 4.3	8.6 ± 3.5	− 5.1** ± 2.5	0.835
Bicarbonate (mmol/L)	32.5 ± 2.2	20.2** ± 3.5	31.7 ± 3.8	19.9** ± 2.3	0.863
Urea nitrogen (mg/dL)	6.9 ± 1.9	91.8** ± 18.8	7.6 ± 2.2	102.6** ± 29.7	0.402
Creatinine (mg/dL)	0.9 ± 0.1	11.7** ± 1.6	0.9 ± 0.1	9.3** ± 1.2	0.005
Potassium (mmol/L)	3.6 ± 0.2	5.8** ± 0.7	3.8 ± 0.2	6.6** ± 0.8	0.084
Calcium (mg/dL)	11.0 ± 0.4	11.1 ± 1.0	10.7 ± 0.4	12.0** ± 1.0	0.108
Phosphorus (mg/dL)	5.9 ± 0.5	16.0** ± 6.2	5.5 ± 0.7	11.4** ± 2.4	0.070
Calcium phosphorus product (mg^2^/dL^2^)	65.5 ± 7.8	173.7** ± 54.4	58.2 ± 7.1	135.1** ± 27.2	0.094
Magnesium (mg/dL)	1.90 ± 0.08	2.03 ± 0.30	2.00 ± 0.29	2.38 ± 0.19	0.058
1,25-dihydroxy Vitamin D_3_ (pg/mL)	643 ± 210	66** ± 18	896 ± 345	81** ± 28	0.213
Whole parathyroid Hormone (pg/mL)	4.1 ± 3.8	92.9** ± 61.6	7.8 ± 8.4	60.1** ± 21.8	0.178
Kt/V	N.A	1.92 ± 0.40	N.A	2.09 ± 0.92	0.640

The data indicate mean ± s.d.

*P < 0.05 and **P < 0.01 vs .Day 0 by Student’s t-test. *P* values between the control group and the treatment group by Student’s t-test were shown in the rightmost column.

### Coronary artery calcification

**Figure 3:**
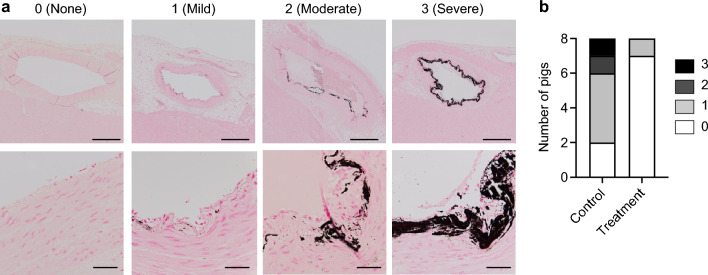
Effects of the alendronate column on coronary artery calcification. Coronary artery calcification was evaluated by grading the severity using von Kossa staining of regular paraffin sections (**a**). Black deposits indicate calcium-phosphate, bar = 500 μm (upper raw) or 50 μm (lower raw). (**b**) Distribution of the calcification score.

Coronary artery calcification correlates positively with circulating CPP levels in hemodialysis patients and independently predicts cardiovascular events and all-cause mortality^19,21^. The severity of coronary artery calcification was graded 0 (none), 1 (mild), 2 (moderate), or 3 (severe) by histological examination with von Kossa staining (Fig. 3a). Calcification was observed in the intimal layer in 6 out of the 8 pigs in the control group, whereas only 1 out of the 8 pigs in the treatment group had mild calcification (*P* = 0.0117 by Chi-square test, *P* = 0.0406 by Fisher’s exact test, Fig. 3b). Calcification was associated with intimal thickening (Fig. 3a), but atheromatous plaques were not observed.

### Vascular endothelial dysfunction

**Figure 4:**
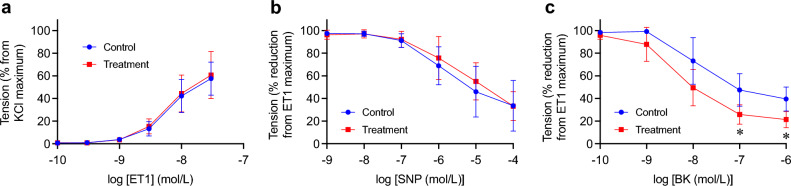
Effects of the alendronate column on vascular endothelial dysfunction. Ex vivo vasoconstriction and vasodilation responses in coronary artery rings obtained from the control group (blue, *N* = 4) and the treatment group (red, *N* = 7) at dissection on Day 28. (**a**) Dose–response curves of vasoconstriction induced by endothelin-1 (ET1). The vascular tension was expressed as a percent of the maximum tension induced by 66.7 mM potassium chloride. (**b**) Dose–response curves of endothelium-independent vasodilation induced by sodium nitroprusside (SNP). (**c**) Dose–response curves of endothelium-dependent vasodilation induced by bradykinin (BK). The coronary artery rings were precontracted with endothelin-1 (30 nM) before treated with SNP or BK. The vascular tension was expressed as a percent reduction from the precontraction induced by endothelin-1. The data represent the mean ± s.d. *P < 0.05 vs the control group by multiple t-test with Holm-Šídák method.

Vascular endothelial dysfunction is associated with end-stage renal disease patients and contributes to the increased risk for cardiovascular complications^22^. To evaluate vascular endothelial function, we measured vasoconstriction and vasodilation responses using isolated coronary artery ring preparation ex vivo. Vasoconstriction responses to endothelin-1 were not different between the control group and the treatment group (Fig. 4a). Vasodilation responses to sodium nitroprusside, which induces endothelium-independent vasodilation, were not different, either (Fig. 4b). However, vasodilation responses to bradykinin, which induces endothelium-dependent vasodilation, were significantly improved in the treatment group (Fig. 4c), indicating that removal of CPPs alleviated vascular endothelial dysfunction.

### Metastatic pulmonary calcification

**Figure 5:**
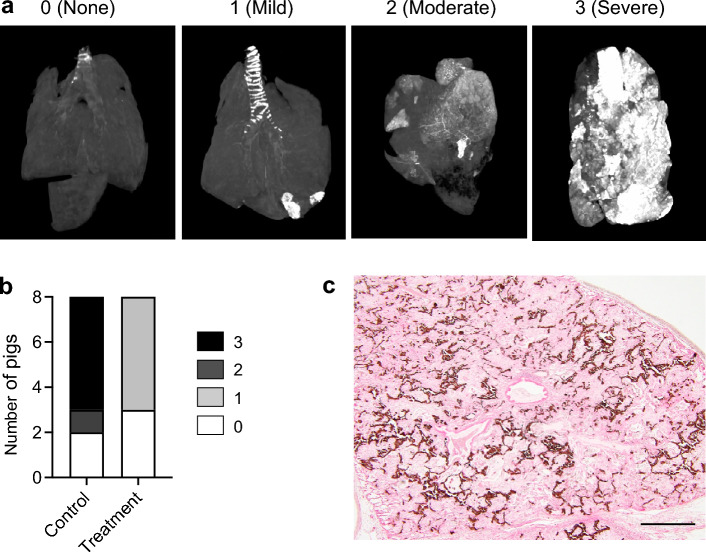
Effects of the alendronate column on metastatic pulmonary calcification. Metastatic pulmonary calcification was evaluated by grading the severity using 3D-CT images of the formalin-fixed lungs (**a**). (**b**) Distribution of the calcification score. (**c**) Von Kossa staining of the lung with severe calcification in the alveolar walls, bar = 500 μm.

Metastatic pulmonary calcification (calcium-phosphate deposition in the lung) was observed in 60–75% of hemodialysis patients at autopsy^23^. The severity of calcification was graded 0 (none), 1 (mild), 2 (moderate), or 3 (severe) by three-dimensional computed tomography (3D-CT) imaging of the formalin-fixed lungs (Fig. 5a). Moderate to severe calcification was detected in 6 out of the 8 pigs in the control group, but not in the treatment group (*P* = 0.0019 by Chi-square test, *P* = 0.0070 by Fisher’s exact test, Fig. 5b). Calcification developed mainly in the alveolar walls as seen in dialysis patients (Fig. 5c)^23^. Despite the severe metastatic pulmonary calcification, the control group had similar arterial blood gas parameters to the treatment group, including pH, bicarbonate, base excess, partial pressure of oxygen and carbon dioxide, although partial pressure of oxygen tended to be lower in the control group than that in the treatment group (*P* = 0.055, Table 1).

### Left ventricular hypertrophy

**Figure 6:**
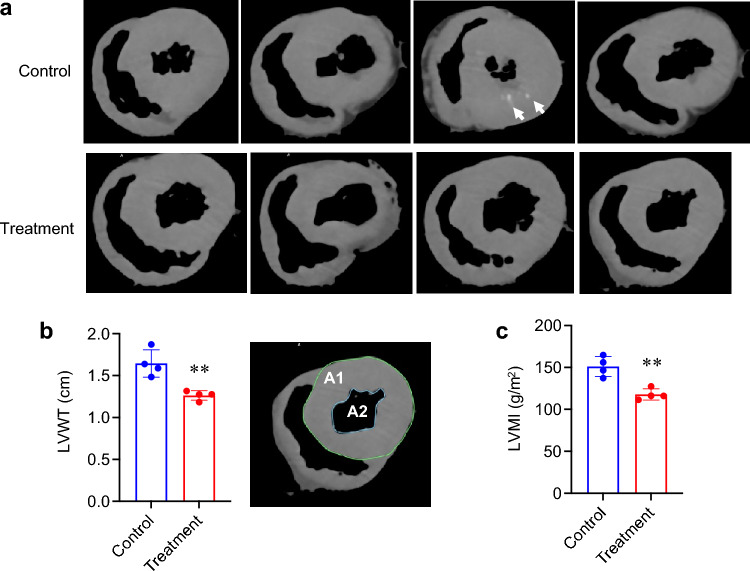
Effects of the alendronate column on left ventricular hypertrophy. Left ventricular hypertrophy was evaluated using 3D-CT images of the formalin-fixed hearts (**a**). Arrows indicate ectopic calcification. (**b**,**c**) The treatment group had significantly greater left ventricular wall thickness (LVWT) and left ventricular mass index (LVMI) than the control group (*N* = 4 for each group, **P < 0.01, by Student’s t test). LVWT was calculated using the following formula: $\sqrt{A1/\pi }- \sqrt{A2/\pi }$, where A1 and A2 represent the area as defined in (**b**).

Left ventricular hypertrophy is commonly observed in hemodialysis patients and identified as a potent risk for cardiovascular mortality in CKD patients^24^. The blood pressure was not different between the treatment group and the control group (Table 1). However, 3D-CT images of the formalin-fixed hearts revealed that the control group had greater left ventricular wall thickness (LVWT) and left ventricular mass index (LVMI) than the treatment group (Fig. 6a,b). Of note, ectopic calcification in the left ventricular wall was observed in one of the pigs in the control group (Fig. 6a).

### Calcification propensity and inflammation

**Figure 7:**
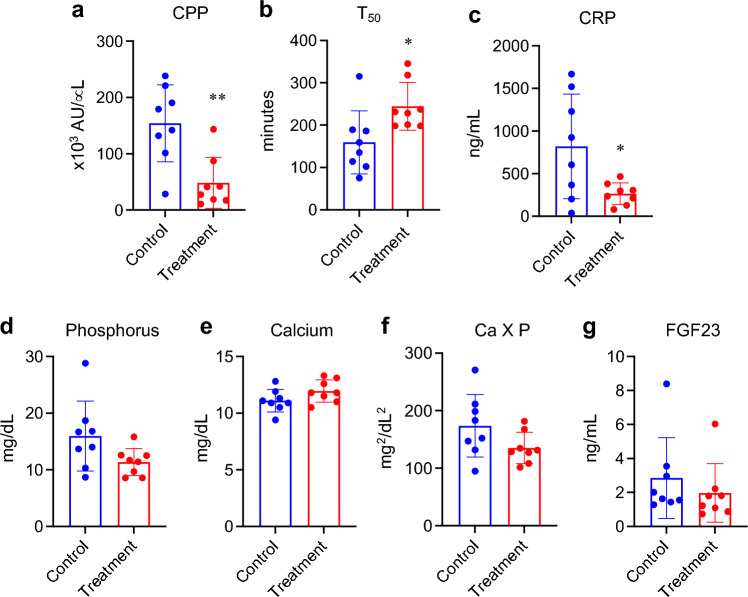
Effects of the alendronate column on blood parameters. Blood samples collected after the hemodialysis session on Day 14 were used for measuring CPP (**a**) and T_50_ (**b**). Blood samples collected before the hemodialysis session were used for measuring CRP (**c**), phosphorus (**d**), calcium (**e**), calcium phosphate product (**f**) and FGF23 (**g**). The data indicate mean ± s.d., *N* = 8 for each group, *P < 0.05 and **P < 0.01 by Welch’s t-test.

A cross-sectional comparison of several blood parameters between the control group and treatment group was performed on the 14^th^ day after the nephrectomy (Day 14), when all the 16 pigs in both groups were alive. The treatment group had lower plasma CPP and higher plasma T_50_, an index of anti-calcifying propensity^25^, than the control group after the hemodialysis session (Fig. 7a,b). The treatment group had lower plasma C-reactive protein (CRP) levels than the control group, indicating that removal of CPPs from the blood alleviated chronic inflammation (Fig. 7c). However, serum levels of phosphorus, calcium, calcium phosphorus product, and fibroblast growth factor-23 (FGF23) were not significantly different between the groups (Fig. 7d–g). The other parameters that did not reach statistically significant difference were shown in Table 1.

A longitudinal comparison of several parameters between Day 0 (before the nephrectomy) and Day 14 (before the hemodialysis session) was performed in each group (Table 1). A decrease in the diastolic blood pressure and an increase in the pulse pressure were observed in the control group, suggesting progression of arterial stiffness. Both groups developed metabolic acidosis characterized by a decrease in bicarbonate, pH, and base excess. Blood urea nitrogen, creatinine, phosphorus, potassium, and whole parathyroid hormone were significantly increased, whereas 1,25-dihydroxyvitamin D_3_ was decreased in both groups, all of which were changes commonly observed in renal failure patients.

### Fetuin-A

**Figure 8:**
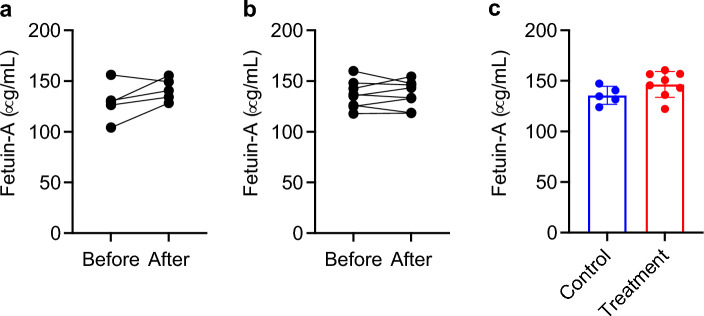
Blood fetuin-A levels. Plasma samples collected before and after the hemodialysis session on Day 22 were used for measuring fetuin-A in the control group (**a**) and the treatment group (**b**). No difference by paired t-test. (**c**) Plasma fetuin-A levels before the hemodialysis session on Day 22 in the control and treatment group. The data indicate mean ± s.d. No difference by Student’s t-test.

Removing CPPs from the circulation means removal of fetuin-A as well. To check if hemodialysis using the alendronate column causes fetuin-A deficiency, we measured plasma fetuin-A levels immediately before and after the hemodialysis sessions on Day 22 and found no significant difference between them both in the control and treatment groups (Fig. 8a,b). No difference in the average fetuin-A levels was observed between the control and treatment groups, either (Fig. 8c). Because fetuin-A is produced and secreted almost exclusively from the liver^26^, plasma fetuin-A levels may not be significantly decreased by removal of CPP2 as long as the liver is healthy.

### Effects of CPP removal in vitro

**Figure 9:**
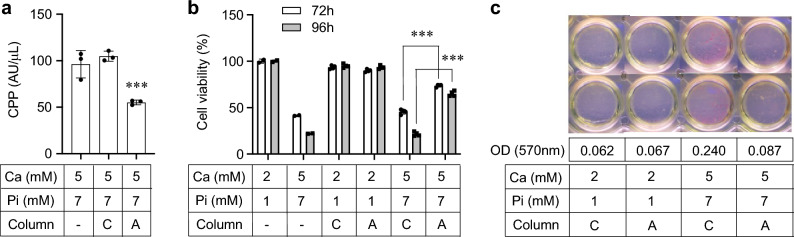
Effects of CPP removal from the medium on cultured vascular endothelial and smooth muscle cells. (**a**) CPP levels in the tissue culture medium. The CPP-containing medium was prepared by increasing the calcium (Ca) and phosphate (Pi) concentration to 5 mM and 7 mM, respectively. CPP levels in the medium were measured before (-) and after filtration through the control column (C) or the alendronate column (A). The data represent the mean ± S.D. *N* = 3 for each group, ***P < 0.0001 vs C by Student’s t-test. (**b**) Effects of CPP removal on cell viability of cultured human vascular endothelial cells (HUVEC). Endothelial cells were cultured in CPP-free medium (the regular growth medium containing 2 mM calcium [Ca] and 1 mM phosphate [Pi]), CPP-containing medium (same as the CPP-free medium except that the [Ca] and [Pi] were increased to 5 mM and 7 mM, respectively), or the flow-through fraction of these media from either the control column (C) or the alendronate column (A) for 72 or 96 h. The data represent the mean ± S.D. *N* = 4 for each group, ***P < 0.0001 by Student’s t-test. (**c**) Effects of CPP removal on calcification of cultured rat vascular smooth muscle cells (A7r5). Smooth muscle cells were cultured in CPP-free medium, CPP-containing medium, or the flow-through fraction of these media from either the control column (C) or the alendronate column (A) for 28 days and then stained with Alizarin red that binds to calcium. The average values of optical density (OD) at 570 nm of the cell extracts were indicated.

To determine whether the effects of the alendronate column on vasculature could be recapitulated in vitro, we cultured vascular endothelial and smooth muscle cells with the CPP-containing medium that had passed through either the alendronate column or the control column. The control column barely removed CPPs from the medium, whereas the alendronate column removed about 50% of the CPPs (Fig. 9a). The flow-through fraction from the alendronate column induced less cell death in endothelial cells (Fig. 9b) and less calcification in smooth muscle cells (Fig. 9c) than that from the control column.

## Discussion

The present study identified CPPs as a causative agent of coronary artery calcification, vascular endothelial dysfunction, metastatic pulmonary calcification, left ventricular hypertrophy, chronic inflammation, and increased mortality in hemodialysis miniature pigs. It should be noted that the alendronate column alleviated these disorders without significantly lowering blood phosphorus levels (Table 1), suggesting that CPPs, rather than phosphorus per se, may be primarily responsible for these disorders. This notion is consistent with our previous tissue culture experiment showing that cell death induced by increasing the phosphate concentration in the tissue culture medium was inhibited by blocking formation of calcium-phosphate crystals^27^. As clinical studies have shown that blood phosphate levels correlated positively with blood CPP levels^2,3^, phosphate lowering therapy by restriction of dietary phosphate intake and administration of phosphate binders, the current standard in clinical care, may improve clinical outcomes of patients with hyperphosphatemia through lowering blood CPP levels. Hyperphosphatemia may contribute to the poor prognosis through facilitating CPP formation, at least in part.

Many prospective clinical trials have been conducted aiming at slowing progression of vascular calcification in CKD patients. Among them, trials using magnesium or sodium thiosulfate consistently gave promising results^28,29^. Although the mechanism of action of sodium thiosulfate remains elusive, magnesium was reported to inhibit the amorphous-to-crystalline phase transition of calcium-phosphate *in vitro*^25^. More recently, a derivative of phytate (SNF472) was shown to slow coronary artery and aortic valve calcification in a randomized phase 2b study^30^. SNF472 also inhibited the amorphous-to-crystalline phase transition *in vitro*^31^. Therefore, these reagents slow progression of vascular calcification probably through inhibiting CPP maturation. In contrast, the alendronate column prevented vascular calcification through removing CPPs from the blood. The different mechanism of action between the alendronate column and magnesium/phytate derivatives has raised the possibility that the combination therapy may provide additive or synergistic benefits for the treatment of vascular calcification.

The present data indicate that removal of CPPs using the alendronate column alleviated intimal thickening (Fig. 3) and endothelial dysfunction (Fig. 4) of coronary arteries. Although the link between hyperphosphatemia and vascular calcification has been well established, reports showing a potential link between hyperphosphatemia, intimal thickening, and endothelial dysfunction are limited. Shuto et al. observed impaired endothelium-dependent vasodilation in rat aorta ring preparations bathed in high phosphate medium ex vivo and in healthy men after loading a high phosphate meal *in vivo*^32^. Jung et al*.* observed independent association between serum phosphate levels and parameters of endothelial dysfunction in hemodialysis patients^33^. These observations can be explained by assuming that hyperphosphatemia may induce endothelial dysfunction through elevating circulating CPP levels and trigger intimal thickening through the mechanism yet to be determined^34^.

The alendronate column is expected to remove secondary CPPs from the blood more efficiently than primary CPPs and CPMs, because crystalline calcium-phosphate binds to alendronate with higher affinity than amorphous calcium-phosphate^3^. In the present study, we measured plasma CPP levels by the gel-filtration method, which primarily quantifies the amount of crystalline calcium-phosphate in secondary CPPs. Thus, the fact that CPP levels determined by the gel filtration method were lower in the blood obtained from the outlet of the alendronate column than those from the inlet (Fig. 1b) indicates that the alendronate column indeed adsorbed secondary CPPs. However, the majority of CPPs circulating in the blood are CPMs and primary CPPs^3^. Despite that, the alendronate column removed CPPs from the blood in an efficient manner. Indeed, we previously demonstrated that magnetic beads conjugated with alendronate pulled down CPPs from fresh plasma samples of hemodialysis patients *in vitro*^3^. It is possible that alendronate may bind to CPMs and primary CPPs, albeit with lower affinity. It is also possible that a part of calcium-phosphate precipitates in primary CPPs undergo the amorphous-to-crystalline phase transition when passing through the hemodialysis circuit and the alendronate column. Foreign surfaces in the extracorporeal circulation circuit activate blood coagulation, which cannot be completely blocked even under anticoagulation. We reported that blood coagulation accelerated the phase transition and secondary CPP formation^3^. Thus, amorphous calcium-phosphate in the primary CPPs may be transformed to crystalline calcium-phosphate in the extracorporeal circulation circuit and captured efficiently by the alendronate column.

The fact that the alendronate column removed secondary CPPs may explain why the treatment group had longer plasma T_50_ values than the control group (Fig. 7b). T_50_ is a blood test to evaluate overall propensity of the blood for secondary CPP formation *in vitro*^25^*.* Briefly, a serum/plasma sample is inoculated with a fixed amount of phosphate and calcium to facilitate secondary CPP formation, and then the turbidity of the sample is monitored over for several hours. A sudden increase in turbidity due to Tyndall effect is observed when the existing CPPs in the sample are agglomerated and matured to large secondary CPPs with diameter of a few hundred nm (Supplementary Fig. S1b). The time interval between the calcium/phosphate addition and the sudden increase in turbidity is defined as T_50_. Therefore, low (short) T_50_ indicates that the blood is rich in “mature” CPPs that can be readily transformed to secondary CPPs. Plasma samples of the control group contain CPMs, primary CPPs, and secondary CPPs (Supplementary Fig. S1c). On the other hand, plasma samples of the treatment group should contain less secondary CPPs than those from the control group, because secondary CPPs have been removed by the alendronate column (Supplementary Fig. S1d). Therefore, the plasma samples from the treatment group require a longer time to yield the large secondary CPPs causing the Tyndall effect than those from the control group, which may explain why T_50_ was increased in the treatment group. Further studies are required to clarify the precise mechanism by which the alendronate column efficiently removes CPPs from the circulation.

Further studies are also needed to clarify the mechanism by which the alendronate column alleviated left ventricular hypertrophy (Fig. 6). Blood pressure was not significantly different between the treatment group and the control group (Table 1). FGF23 was reported to act directly on cardiomyocytes and induce hypertrophy independently of its obligated co-receptor Klotho^35^. However, plasma FGF23 levels was not significantly different between the two groups (Table 1). Accumulating evidence has indicated that activation of Toll-like receptor-4 (TLR4) signaling induces hypertrophic remodeling of the heart^36^. We previously reported that CPPs bound to TLR4 and activated NFκB signaling in renal proximal tubular cells^27^. A hypothesis that CPPs may directly act on cardiomyocytes through TLR4 to induce hypertrophy is worth testing.

Insoluble biological materials (lipids and calcium-phosphate) form complexes with specific serum proteins (apoproteins and fetuin-A) and are dispersed in the blood as colloidal particles (lipoproteins and CPPs) to be transported eventually to their storage organs (fat and bone). However, when mistargeted to vasculature, lipoproteins induce atherosclerosis^5^. The present study has shown that CPPs induce vascular calcification. Two distinct forms of arteriosclerosis, atherosclerosis and vascular calcification, can be sublated as a disorder caused by mistargeting of colloids to vasculature.

## Methods

### Ethical statement

All animal experiments were performed based on relevant guidelines/regulations and ARRIVE (Animal Research: Reporting of in Vivo Experiments) guidelines. All animal experimental protocols were approved in advance by the institutional animal care and use committee from Nihon Bioresearch Center certified as an institute in compliance with GLP (Good Laboratory Practice) by Pharmaceuticals and Medical Device Agency, Japan, and carried out at Nihon Bioresearch Center. All methods are reported based on ARRIVE guidelines.

### Miniature pigs

Nippon Institute for Biological Science (NIBS) miniature pigs (10 ± 2-month-old males) weighing 23.2 ~ 31.9 kg were housed individually in a stainless cage placed in a room controlled for temperature (24 ± 4 °C), humidity (55 ± 25%), and lighting (12-h light/dark cycle). After 10 days of quarantine and acclimatization, the miniature pigs were given 400 g/day of regular swine diet (MP-A, Oriental Yeast Co., Ltd.) once a day (16:00–18:00) with free access to tap water. The diet was supplemented with 0.3 g/kg of inorganic phosphate to accelerate ectopic calcification.

### Bilateral nephrectomy

After sedation with intramuscular injection of atropine sulfate (0.05 mg/kg), medetomidine hydrochloride (0.05 mg/kg), and midazolam (0.5 mg/kg), miniature pigs were anesthetized with nitrogen monoxide (N_2_O:O_2_ = 1:1) and isoflurane (0.5 ~ 1.5%) through a tracheal intubation tube. A hemodialysis catheter (Blood Access UK Catheter Kit, Nipro Medical, Osaka, Japan) was inserted into the internal jugular vein and locked with heparinized saline. After laparotomy and ligation of renal arteries, renal veins, and ureters, both kidneys were excised.

### CPP adsorption column

The CPP adsorption column was filled with 120 ml of porous cellulose beads with an average diameter of 460 μm covalently conjugated with the amine group of alendronate (the alendronate column). Briefly, cellulose beads were incubated with epichlorohydrin under an alkaline condition at 40 °C for 2 h to introduce epoxy groups on the surface of the beads. The epoxidized cellulose beads were then incubated with alendronate at 50 °C for > 5 h to introduce a covalent bond between the epoxy group on the beads and the amine group of alendronate by nucleophilic reaction. The control column was filled with the same volume of cellulose beads without alendronate conjugation.

### Extracorporeal circulation

On the 4^th^ day after bilateral nephrectomy, miniature pigs were subjected to extracorporeal circulation through the hemodialysis catheter using the pump of a hemodialysis device (NCV-10, Nipro, Osaka, Japan) and a blood circuit set (Sureflow N, Nipro). Either the alendronate column or the control column was inserted into the circuit. Blood sampling was performed from the inflow and the outflow of the column 30 min after starting extracorporeal circulation. After the blood sampling, the pigs were euthanized by rapid intravenous injection of potassium chloride under anesthesia.

### Hemodialysis

Bilateral nephrectomy and hemodialysis catheter placement were performed on Day 0. On Day 2, the nephrectomized miniature pigs were subjected to hemodialysis under awake condition using hemodialysis devices (NCU-12 or NCV-10, Nipro, Osaka, Japan), dialyzers (FB-90P β ECO, Nipro), dialysates (AK-SOLITA DL, AY Pharmaceuticals Co., Ltd., Tokyo, Japan), and blood circuit sets (Sureflow N, Nipro) every other day thereafter through Day 28. The composition of dialysate was sodium 140, potassium 2.0, calcium 3.0, magnesium 1.0, chloride 111, bicarbonate 25, acetate 10 in mEq/L, and glucose 1.0 g/L. The composition of dialysate was not changed throughout the study. Either the alendronate column (the treatment group, *N* = 8) or the control column (the control group, *N* = 8) was inserted upstream of the dialyzer in every hemodialysis session. The dialysis condition was as follows: blood flow rate, 150 ml/min; dialysate flow rate, 300 ml/min; duration, 5 h. The anti-coagulation was implemented using dalteparin. Dalteparin sodium was administered by bolus intravenous injection (50 unit/kg) immediately before starting hemodialysis, followed by continuous injection (100 unit/h) during the dialysis session. After every dialysis session, the miniature pigs were administered intravenously with erythropoietin (1,000 units of Espo, Kyowa-Kirin, Japan,), saccharated ferric oxide (20 mg of Fesin, Nichi-iko Pharmaceutical, Japan), and ampicillin (1 g of Viccillin, Meiji Seika Pharma, Japan). In addition, they were administered via gastric tube with potassium-binding resin (9 g of Kayexalate drysyrup, Torii, Japan) and laxative (20 ml of D-sorbitol oral solution 75%, Kowa, Japan) from Day 8 through Day 28, and anti-ulcer drug (20 mg of Gaster, Astellas, Japan) every day.

### Blood sampling and analyses

Blood sampling was performed from the hemodialysis catheter placed in the internal jugular vein on Day 14. Immediately after sampling using a syringe, the blood was transferred to a heparinized tube and placed on ice. Within 15 min after sampling, plasma was separated by centrifugation (4 °C, 1600 g, 15 min). The plasma sample was aliquoted, snap-frozen in liquid nitrogen, and stored at − 80 °C before the biochemistry, CPP, and T_50_ assay. Plasma fetuin-A and FGF23 levels were measured using Pig AHSG/Fetuin-A ELISA Kit (LSBio, Seattle, WA) and Pig Intact Fibroblast Growth Factor-23 ELISA Kit (Kamiya Biomedical Company, Tukwila, WA), respectively. The hemodialysis efficiency was evaluated by Kt/V, which was calculated by the Daugirdas formula: -Ln(Ct/Co-0.008t) + (4–3.5Ct/Co) × △BW/(BW), where Ct = blood urea nitrogen (BUN) after dialysis (mg/dL), Co = BUN before dialysis (mg/dL), t = dialyzing time (hours), △BW = difference in body weight between before and after the dialysis (kg), BW = body weigh after the dialysis (kg). Plasma CPP levels were quantified by the gel-filtration method^3^. Briefly, a fluorescent probe (OsteoSense^®^, a bisphosphonate conjugated with an infrared dye, MW = 1.5 kDa) was added to a plasma sample and incubated at 25 °C to let the probe bind to calcium-phosphate in CPPs. Because crystalline calcium-phosphate has higher binding affinity to bisphosphonate than amorphous calcium-phosphate, the probe bound predominantly to CPPs containing crystalline calcium-phosphate, namely secondary CPPs. One hour later, the sample was applied to a gel-filtration spin column (molecular weight cut-off 40 kDa) to remove excess unbound probe. The fluorescent intensity of the flow through fraction containing CPPs was defined as the CPP level, which primarily reflected the amount of crystalline calcium-phosphate in the secondary CPPs. Plasma T_50_ values were determined by the method reported previously in human sera^25^, except that the amount of calcium added to the plasma samples was reduced to one-half (the final concentration at 5 mM).

### Ex vivo measurement of coronary artery tension

The isolated circumflex coronary artery was cut into 2 mm ring segments and bathed in Krebs–Henseleit buffer (KH buffer) aerated with 95% O_2_ and 5% CO_2_ at 37 °C. The arterial rings were mounted on an isometric transducer (TB-611 or TB-612, Nihon Kohden, Japan) at the resting tension of 2 g. After equilibration for 30 min, the KH buffer was replaced with high potassium buffer (KH buffer containing 66.7 mM KCl) to measure the maximum isometric contraction. After washing with HK buffer, endothelin-1 (Peptide Institute Inc., Japan) was added to the bathing HK buffer to measure dose-dependent contraction and then treated with bradykinin (Peptide Institute Inc., Japan) or sodium nitroprusside (Fujifilm Wako Pure Chemical Co., Japan) to record endothelium-dependent and independent relaxation, respectively.

### Three-dimensional computed tomography (3D-CT)

The formalin-fixed heart and lung were subjected to 3D-CT imaging using Somatom Definition AS + (Siemens Healthineers Japan). Left ventricular wall thickness (LVWT) was calculated as shown in Fig. 6b. Left ventricular mass was calculated by the area-length method. LVMI was calculated by dividing the left ventricular mass with the body surface area.

### Cell culture experiments

Human umbilical vein endothelial cells (HUVEC) and rat aortic smooth muscle cells (A7r5) were purchased from PromoCell (Heidelberg, Germany) and ATCC, respectively, and cultured in respective growth medium recommended by the providers. CPPs were generated in vitro by increasing the calcium and phosphate concentration to 5 mM and 7 mM, respectively, in Dulbecco's Modified Eagle's Medium (DMEM) supplemented with 10% fetal bovine serum (FBS) and incubating at 37 °C for 24 h. This medium was defined as “CPP-containing medium”. DMEM supplemented with 10% FBS contained approximately 2 mM calcium and 1 mM phosphate, but did not give rise to CPPs detectable by the gel-filtration method, thus designated as “CPP-free medium”. The CPP-containing medium and the CPP-free medium were filtrated through the alendronate column or the control column. For cell viability assay, the flow-through fractions were mixed with the equal volume of the growth medium for HUVEC, applied to confluent HUVEC for 72 or 96 h, and subjected to MTT assay (Promega, Madison, WI) according to the manufacturer’s protocol. For calcification assay, the flow-through fractions were mixed with the equal volume of the growth medium for A7r5 and applied to confluent A7r5 cells. This procedure was repeated every 3 days. Four weeks later, the A7r5 cells were fixed with methanol and stained using a calcified nodule staining kit with alizarin red S (Cosmo Bio Co., Ltd., Tokyo,Japan). The bound alizarin red S was eluted with 10% (w/v) cetylpyridinium chloride and quantified by measuring absorbance at 570 nm.

### Statistics

Quantitative data were expressed as mean ± standard deviation except when stated otherwise. Statistical analyses were performed using GraphPad Prism 9.

### Supplementary Information

Supplementary Information.

## Data Availability

The data that support the findings of this study are available from the corresponding author upon reasonable request.
