# Supplementary material for: Removal of calciprotein particles from the blood using an adsorption column improves prognosis of hemodialysis miniature pigs
Source: Sci Rep. 2023 Sep 12;13:15026. doi: 10.1038/s41598-023-42273-0 (PMC10497634; doi:10.1038/s41598-023-42273-0)
Supplement: Supplementary file 1 — Supplementary Information. [file 41598_2023_42273_MOESM1_ESM.pdf]

Supplementary material

Removal of calciprotein particles from the blood using an adsorption column improves prognosis of hemodialysis miniature pigs

Marina Miura[1]†, Yutaka Miura[1]†, Yoshitaka Iwazu[1,2,3], Hideyuki Mukai[1], Takahiro Sugiura[4], Yuji Suzuki[4], Masami Kato[4], Mayumi Kano[4], Daisuke Nagata[3], Kazuhiro Shiizaki[1], Hiroshi Kurosu[1], & Makoto Kuro-o[1] *

[1]*Division of Anti-aging Medicine, Center for Molecular Medicine,*
[2]*Department of Clinical Laboratory Medicine,*
[3]*Division of Nephrology, Department of Internal Medicine,*
Jichi Medical University, Tochigi, Japan.
[4]*Nihon Bioresearch Inc., Gifu, Japan.*

† These authors contributed equally to this work.

*Correspondence:
Makoto Kuro-o, M.D., Ph.D.
Division of Anti-aging Medicine, Center for Molecular Medicine,
Jichi Medical University,
3311-1 Yakushiji, Shimotsuke, Tochigi 329-0498, Japan.
Email: mkuroo@jichi.ac.jp

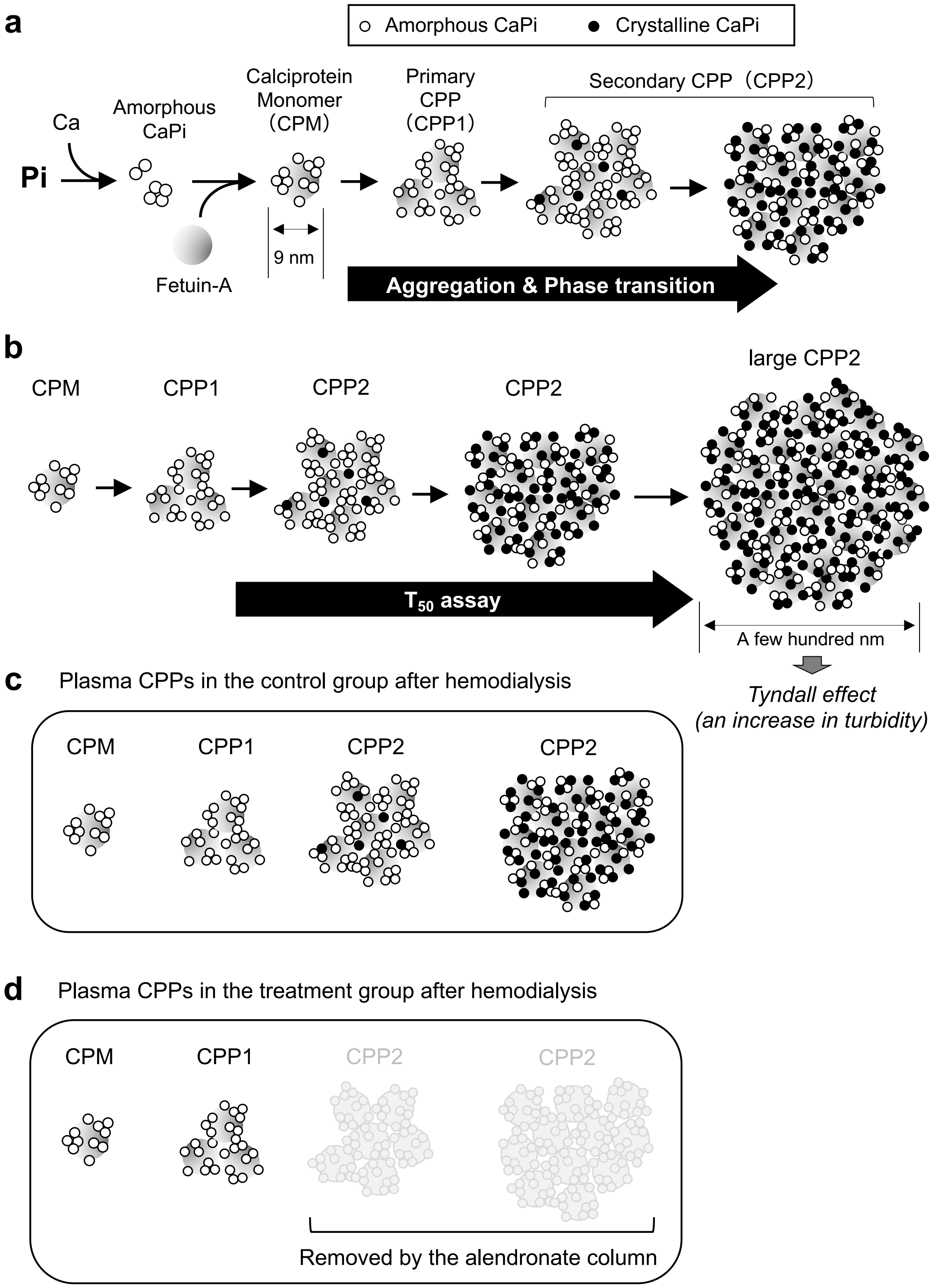

Supplementary Fig. 1| Schematic diagrams for formation and maturation of CPPs.

a, Formation and maturation of CPPs are not a biological process that requires live cells or tissues, but a physicochemical phenomenon that progresses spontaneously over time in a

solution containing calcium (Ca), phosphate (Pi), and fetuin-A under certain conditions are met. CaPi, calcium-phosphate, CPM, calciprotein monomers, CPP1, primary CPPs, CPP2, secondary CPPs. **b**, In the T_{50} assay, calcium and phosphate were added to the plasma samples to increase their concentration by 5 mM and 6 mM, respectively. In addition, the plasma samples were diluted by a factor of 2.5 with buffer. The increase in calcium and phosphate concentration to a supraphysiological range and the decrease in fetuin-A concentration by dilution accelerated formation of CPP2 large enough to increase turbidity of the samples by the Tyndall effect. **c**, A schematic representation of CPPs in plasma samples from the control group. CPP2 were not removed by regular hemodialysis. **d**, A schematic representation of CPPs in plasma samples from the treatment group. CPP2 were removed by the alendronate column. Therefore, CPPs in the plasma samples from the control group (**c**) can mature more quickly into large CPP2 than those from the treatment group (**d**)., which explains why removal of CPP2 by the alendronate column extended T_{50} (Fig. 7b).
